# Effects of chronic cocaine in rat C6 astroglial cells

**DOI:** 10.3892/ijmm.2012.1038

**Published:** 2012-06-20

**Authors:** RAMESH B. BADISA, CARL B. GOODMAN

**Affiliations:** College of Pharmacy and Pharmaceutical Sciences, Florida A&M University, Tallahassee, FL 32307, USA

**Keywords:** astroglial cells, brine shrimp larvae, chronic cocaine, nitric oxide

## Abstract

Investigations with astroglial cells carry equal importance as those with neurons in drug abuse studies. The present study was aimed to investigate the effect of chronic cocaine administration on cell viability, nitric oxide (NO) production, general respiratory status of mitochondria and total protein levels in rat astroglioma cells after 24 h of treatment. In addition, the effect of cocaine was assessed for 24 h on brine shrimp larvae in order to study their sensitivity to the drug. It was observed that cocaine caused a significant dose-dependent decrease in astroglial cell viability with an LC_50_ of 4.717 mM. It was found that cocaine did not induce or inhibit NO production in the cells. Evaluation of mitochondrial dehydrogenase activity in terms of formazan production in astroglial cells indicated that cocaine significantly interfered with the general respiratory status of mitochondria with an ED_50_ of 6.153 mM. Furthermore, cocaine was shown to deplete the total protein levels in the cells with an ED_50_ of 5.435 mM. *In vivo* study with brine shrimp larvae showed that these larvae were highly sensitive to cocaine with an ED_50_ of 2.41 mM. In summary, our findings suggest that cocaine-induced cytotoxicity in the cells was non-specific. The cumulative effect arising from the significant loss of respiration and total cellular proteins is the cause of astroglial cell death.

## Introduction

The usage of drug abuse substances has increased exponentially during the last decade and has become a more serious problem. Cocaine is one of the oldest known psychoactive drugs in the world. For centuries, people in South America (Bolivia, Peru) and other parts of the world chewed the leaves of *Erythroxylon coca* Lam or *Erythroxylon novogranatense* Hieronymus of the Erythroxylaceae family to treat headaches, toothaches and other types of body pain. Because cocaine acts on the CNS as a psychostimulant, its possession, consumption or distribution for non-medicinal purposes is considered illegal in most countries. Present sestimations indicate that one in six Americans has consumed it by the age of 30 years (1).

Research studies indicate that cocaine is toxic to cells within (2–4) or outside the CNS (5–7). In the CNS, cocaine causes gradual loss or dysfunction of several cells simultaneously. However, major attention has been given to neurons alone due to the perception that non-neuronal cells, like astroglial cells, were only passive bystanders in the process of synaptic transmission. However it has been shown that astroglial cells, which are present throughout the CNS, are not just bystanders but important participants in signal transmission. Besides outnumbering the neurons by 10 to 1 (8) in the adult brain, the astroglial cells are critical for neuronal survival and maintenance of fundamental patterns of circuits (9,10). Abundance of astroglial cells may suggest that they could be the earliest targets of cocaine toxicity. Because astroglial cells have an inducible-form of nitric oxide synthase (11), the malfunction of these cells with cocaine exposure could release excessive nitric oxide (NO) as a consequence of inflammation. This in turn may contribute to Parkinson’s disease (12), schizophrenia, or Alzheimer or other diseases. While studies on the cells outside of CNS have demonstrated the inhibitory role of cocaine on NO release (7), not many reports are available on the NO production in astroglial cells of the CNS.

The aim of the present study was to discern the influence of chronic cocaine on the viability, NO production, general respiratory status of the mitochondria, and total protein levels in astroglial cells. We employed rat C6 astroglioma cells in this study based on our earlier reports (3). Cocaine effects were further explored in a simple biological system, brine shrimp, under *in vivo* conditions to understand if cocaine toxicity is of the specific or non-specific type. The interesting aspect of this assay is that the toxicity of any water-soluble compound can be evaluated. Brine shrimp larvae, also called sea monkeys, are small crustaceans, about 1 mm in size and offer several advantages of testing many compounds. Since these larvae have relatively simple nervous systems, the interaction between cocaine and shrimp may provide an opportunity to monitor the behavior of live shrimp larvae directly.

## Materials and methods

### Materials

RPMI-1640, FBS, penicillin/streptomycin sulfate, amphotericin B, PBS and L-glutamine were purchased from Mediatech (Herndon, VA, USA). Cocaine hydrochloride, crystal violet, L-glutaraldehyde, trypan blue, sodium nitroprusside, sulfanilamide, NED, phosphoric acid, and EDTA were supplied by the Sigma Chemical Co. (St. Louis, MO, USA). The CellTiter 96 AQueous One Solution Reagent kit was purchased from Promega (Madison, WI, USA). Dried brine shrimp cysts and instant ocean sea salt were purchased from a local pet shop. All other routine chemicals were of analytical grade.

### Preparation of sample

The stock (1 M) as well as working stocks (80–180 mM) of cocaine hydrochloride in all our studies were always prepared fresh in PBS just prior to assays and applied at different concentrations to astroglial cultures in a minimum volume (5 μl) to prevent pH alterations.

### Cell culture studies

The CNS-derived rat C6 astroglial cell line (CCL-107) was purchased from the American Type Culture Collection (Rockville, MD, USA) and maintained as an adherent monolayer culture in complete RPMI-1640 (modified) medium, 2 mM L-glutamine, 10% (v/v) FBS, 100 U/ml penicillin, 100 μg/ml streptomycin sulfate and 0.25 μg/ml amphotericin B. Cells were grown in a humidified atmosphere of 95% air, 5% CO_2_ at 37°C in an incubator, and sub-cultured twice a week. For cytotoxic studies, the culture was harvested by treating with 0.05% EDTA in PBS for 2 min or less, resulting in a single cell suspension. Cell count was assessed by 0.4% trypan blue dye exclusion assay on a hemocytometer under a light microscope. Dye-stained cells (blue) were counted as dead, while dye-excluded cells were counted as viable. The actual cell numbers were determined by multiplying diluted times compared with initial cell numbers. Cell viability always exceeded 90%. Cells were diluted in complete RPMI-1640 medium, and then seeded in culture plates for the experiments.

### Treatments with cocaine

The cytotoxic studies were performed in 96-well microtiter plates. The cells were seeded at a starting density of 2×10^4^ cells/well in a total volume of 195 μl growth medium supplemented with 10% FBS. The cells were then allowed to adhere to wells in the incubator prior to drug exposure. Cells which were typically about 60–70% confluent were treated at six different concentrations (2–7 mM) of cocaine in a final volume of 5 μl. Controls and the treated samples were always present in different wells of the same 96-well microtiter culture plates. These plates were incubated for 24 h continuously without further renewal of growth media in a 5% CO_2_ at 37°C in an incubator. The period of incubation was selected based on the cell doubling study (3).

### Evaluation of cytotoxicity

Cytotoxicity of cocaine was evaluated by the dye uptake assay using crystal violet as previously described (13). The average absorbance values of controls were taken as 100% cell viability. Absorbance was measured at 540 nm in a microplate reader (Bio-Tek Instruments, Inc., Wincoski, VT, USA). From the treated and control absorbance values, percent cells killed were determined by the following equation: [1-(T/C)] x 100, where T is the average absorbance value of treated cells, and C is the average absorbance value of control cells.

### Nitric oxide assay

Astroglial cells (2×10^4^ cells/well) were seeded in 96-well titer plates in media lacking phenol red. The next day, cells were treated with cocaine at various concentrations (0, 2 and 3 mM) for 24 h. At the end of incubation, 50 μl of media was transferred into a new 96-titer plate and mixed with an equal volume of Griess reagent (1% sulfanil-amide/0.1% NED in 5% phosphoric acid) followed by 10 min incubation in the dark. The absorbance readings at 546 nm were measured in a microplate reader. Results are presented as nmoles of nitrite per 2×10^4^ cells. A standard curve was generated by using various concentrations of sodium nitrite (25–400 μM).

### General respiratory status assay

Mitochondrial respiratory activity was evaluated as per earlier studies (4,14). In 96-well titer plates, 5×10^3^ cells/well were seeded in the complete media. Next day, cells were treated with different concentrations of cocaine (2–7 mM) for 24 h. Three hours prior to the end of incubation, 10 μl of MTS (Promega, Madison, WI, USA) was added to each well and the titer plates were read in a plate reader at 490 nm.

### Total cellular protein measurement

The role of cocaine at different concentrations (2–7 mM) on the total protein levels was assessed in 96-well titer plates (2×10^4^ cells/well) after 24 h incubation as per the earlier method (15). After a fixation step with 0.25% glutaraldehyde for 30 min at room temperature, 20 μl of 0.1% Triton X-100 in dPBS was added per well, followed by incubation at 37°C for 1 h to lyse the cells. Then 80 μl of BCA protein assay reagent (Pierce, Rockford, IL, USA) was added per well and incubated at 37°C for 30 min. The absorbance at 562 nm was measured using a microplate reader.

### In vivo lethality test by brine shrimp bioassay

The brine shrimp cysts (*Artemia salina*) were seeded for rehydration on the surface of artificial seawater, prepared with 1.9% salt mixture (Instant Ocean; Aquarium Systems, Inc., Mentor, OH, USA) in deionized water in a tank under constant illumination at room temperature (22–28°C). The lethality assay was performed in triplicate vials each with ten larvae at various cocaine doses (2, 4 and 6 mM) for 24 h as described by Meyer *et al* (16) and modified by McLaughlin (17). Untreated shrimp larvae in artificial seawater served as controls.

### Statistical analysis

The experimental results are presented as mean ± standard error mean (SEM). The data were analyzed for significance by one-way ANOVA and then compared by the Dunnett’s multiple comparison tests using the GraphPad Prism Software, version 3.00 (GraphPad Software, Inc., San Diego, CA, USA). The test value of P<0.05 was considered significant. All graphs were plotted between the concentration of cocaine and the percentage of cell viability using the above software program. The LC_50_ or ED_50_ values, representing the millimolar concentration of cocaine needed to show 50% response was determined from the graphs (18).

## Results

### Dose-dependent cytotoxicity of cocaine

**Figure 1.**
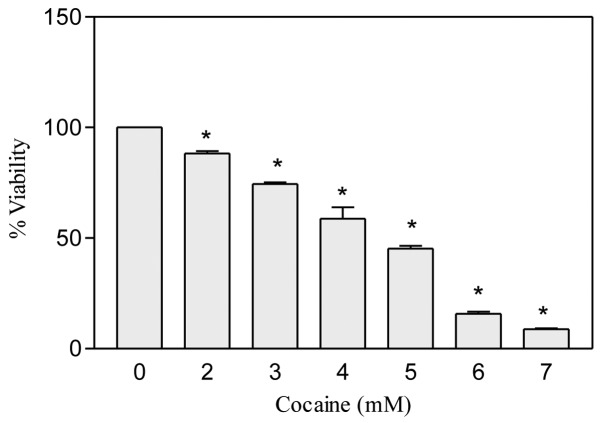
Effect of cocaine on astroglial cell viability. The cells were seeded in 96-well plates with complete RPMI-1640 medium containing 10% FBS and were treated with various concentrations of cocaine for 24 h. Data were represented as mean ± SEM (n=12, ^*^P<0.01, highly significant in comparison to control, one-way ANOVA, Dunnett’s multiple comparison test).

Cocaine concentrations less than 1 mM did not show any apparent effect on the cell viability after 24 h. Therefore after re-adjustment, a total of six different concentrations of cocaine (2–7 mM) were tested in astroglial cells. It was observed that in comparison to the control, cocaine treatment caused a significant (P<0.01, n=12) dose-dependent decrease in the viability of cells (Fig. 1). The cocaine lethal concentration, LC_50_, where 50% cells were killed, was found to be 4.717 mM.

### Cocaine does not stimulate or inhibit NO production

**Figure 2.**
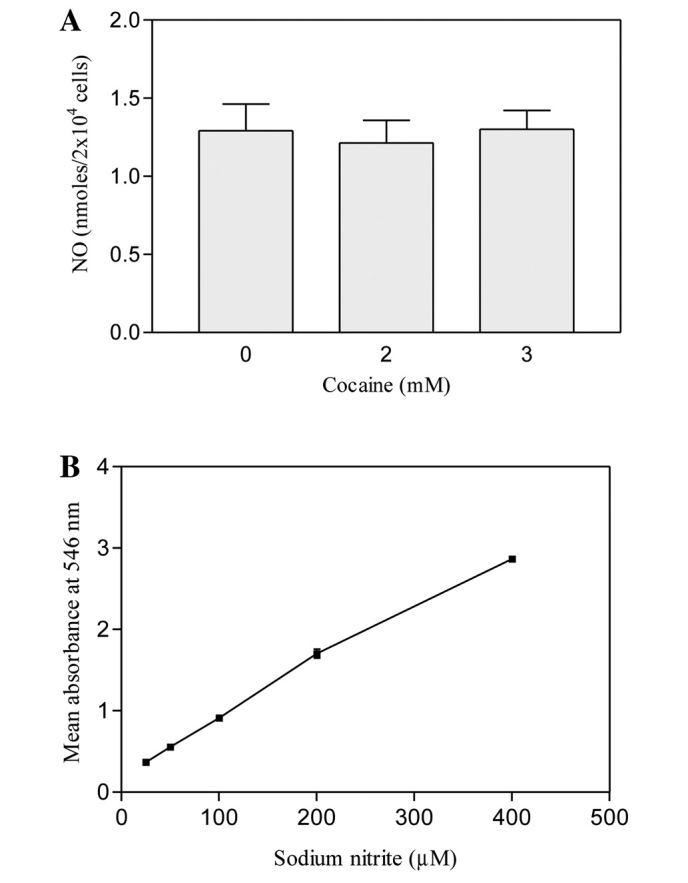
(A) Nitric oxide production in cocaine-treated astroglial cells. The cells were seeded in 96-well plates with complete RPMI-1640 media lacking phenol red, containing 10% FBS and treated with 2 or 3 mM cocaine for 24 h. Nitric oxide was detected with Griess reagent. Data are presented as mean ± SEM (n=15, P>0.05, insignificant in comparison to control, one-way ANOVA, Dunnett’s multiple comparison test). (B) Standard curve of sodium nitrite (25 to 400 μM).

NO is unstable at physiological pH (7.4); its interaction with air eventually leads in the formation of a more stable nitrite in the media. This can easily be detected by Greiss reagent, which has the capacity to react with as low as 2.5 μM nitrite in the assay system to give a deep purple color. In our study, we did not challenge the astroglial cells with bacterial lipopolysaccharide or γ interferon during the treatment period because astroglial cells have iNOS (11) and we wanted to know if cocaine induces this enzyme for excessive NO production. It was found that in comparison to the control, cocaine at 2 or 3 mM concentration did not stimulate or inhibit NO production significantly in the cells (P>0.05, n=15) after 24 h (Fig. 2A). On the other hand, the standard curve of sodium nitrite exhibited a linear dose-dependent response in NO production (Fig. 2B).

### Decrease in mitochondrial respiratory status

**Figure 3.**
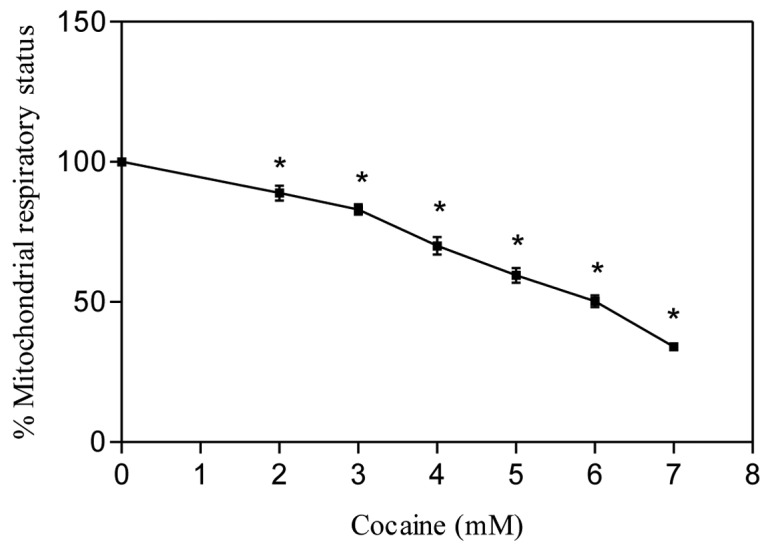
Effect of cocaine on mitochondrial respiratory status in astroglial cells. Cells were seeded in 96-well plates and treated with various concentrations of cocaine for 24 h. Data are presented as means ± SEM (n=12, ^*^P<0.01, highly significant in comparison to control, one-way ANOVA, Dunnett’s multiple comparison test).

The dehydrogenase enzyme located in the mitochondrial membrane reduces the tetrazolium compound of MTS into formazan. Changes in the dehydrogenase activity alter the amount of formazan production in the cells (19). Thus, measurement of total formazan in cells reflects the general respiratory status of mitochondria in the presence of drugs (3,14). In our study, treatment with cocaine at increasing concentrations (2–7 mM) for 24 h caused a significant (P<0.01, n=12) decrease in the mitochondrial respiratory status of astroglial cells (Fig. 3). This was evidenced as a progressive decrease in the amount of formazan production observed in cocaine-treated cells compared to the control. The ED_50_ of cocaine, where 50% loss of mitochondrial activity was observed, was found to be about 6.153 mM.

### Depletion in total cellular proteins

**Figure 4.**
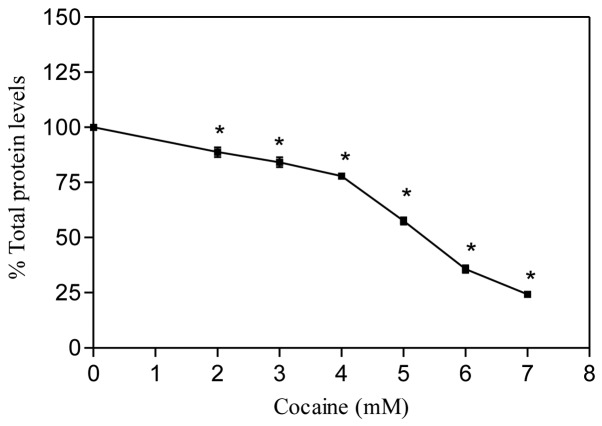
Effect of cocaine on total cellular protein in astroglial cells. Cells were seeded in 96-well plates and treated with various concentrations of cocaine for 24 h. Data are presented as mean ± SEM (n=6, ^*^P<0.01, highly significant in comparison to control, one-way ANOVA, Dunnett’s multiple comparison test).

The BCA reagent is usually used to measure the protein content directly in cells or in lysates. When total protein levels are measured directly in the cells, they may serve as a measurement of cell growth (15). Another way of understanding this assay is to determine how pharmacological treatments affect the total protein levels in the cells. In the present study, we quantified the total protein levels directly in astroglial cells with cocaine treatment after 24 h. The results are presented in Fig. 4. It was observed that in comparison to the control, cocaine treatment significantly decreased the total protein levels (P<0.01, n=6) in a dose-dependent manner. The cocaine ED_50_, where 50% total protein decreased, was found to be 5.435 mM.

### Cocaine toxicity to shrimp larvae

**Figure 5.**
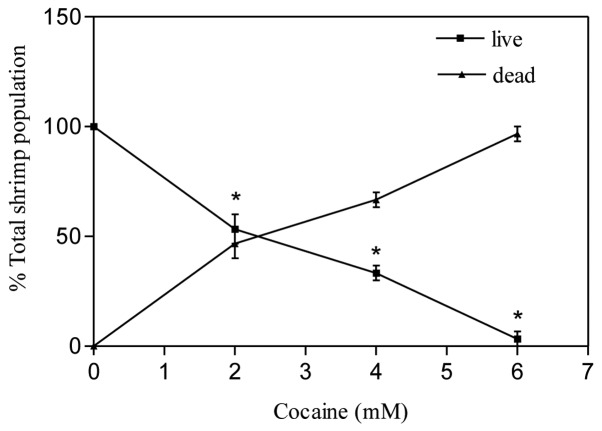
Effect of cocaine on brine shrimp larvae. The assay was carried out at 0, 2, 4 and 6 mM cocaine. After 24 h, the live shrimp were counted in each vial. Data are presented as mean ± SEM (n=3, ^*^P<0.01, highly significant in comparison to control, one-way ANOVA, Dunnett’s multiple comparison test).

The brine shrimp assay is an inexpensive bench-top assay with several advantages in the study of elementary toxicity of drugs in cancer (3,17) and neuropharmacology research. One of the primary benefits of this assay is that several shrimp larvae can be tested at the same time with a drug compound. In our study, under normal circumstances (control), the brine shrimp larvae were seen swimming actively by rhythmic movement of appendages found on their heads in all directions in the vials. Cocaine treatments of shrimp larvae for up to 2 h at 2, 4 and 6 mM did not cause any visual change in their movements. After several hours (12–18 h), even though all larvae were alive, they appeared to be weak in terms of less periodicity in their rhythmic movements in comparison to the control. Continuous exposure to cocaine for 24 h caused significant (P<0.01) death of larvae in comparison to control. The effective dose, ED_50_, where 50% shrimp mortality occurred, was found to be 2.41 mM (Fig. 5).

## Discussion

Cocaine concentrations tested in this study were comparable to earlier reports on various cells (3,4,20–23). In addition, these concentrations were within the acceptable limits of drug addicts considering the fact that its level in the blood of drug addicts is often underestimated due to drug tolerance, frequent drug usage (24) and hydrolysis by esterases (25).

Under our experimental conditions, the LC_50_ was found to 4.717 mM cocaine at 24 h. This value was close to that in previous reports (3,23). It may be noted that FBS contains esterases (26), which could degrade cocaine during chronic cytotoxic incubations. Similarly, cocaine is also known to be hydrolyzed non-enzymatically over a period of time (27,28). In either case, the production of non-toxic pharmacological metabolites, like ecgonine methyl ester, benzyolecgonine or ecgonine, could decrease cocaine toxicity to the cultured cells. Comparison of dose-dependent cell viability data (Fig. 1) indicates that cocaine significantly exacerbated cell death at higher concentrations. This observation suggests that various esterases in FBS did not have a major role on cocaine degradation, and thus imply that astroglial cell death in this study was due to cocaine toxicity.

Recently, there has been an increased interest in the usage of several natural herbs for various health-promoting benefits, such as wound healing and others. One of the popular herbal drugs is Triphala. Its usage has been shown to increase immunity and longevity (29,30). Cocaine toxicity to various cells has been demonstrated (3,4,20–23), while the alleged health benefits of Triphala have been extensively reported (29–31). We were interested in exploring whether Triphala could mitigate the cocaine-induced toxicity to astroglial cells. We did not find any significant protective potential of Triphala (0.2 mg/ml) at any cocaine concentration after 24 h (data not shown). This observation suggests that the target sites of cocaine and Triphala in astroglial cells were different. At higher concentrations (>0.2 mg/ml) Triphala caused cell death (data not shown).

Overproduction of NO, which is a diffusible neurotransmitter in the CNS, contributes to various pathological conditions, including arthritis, Parkinsonism (32), Alzheimer’s and rheumatism (33,34). Previous studies have indicated that astroglial cells have an inducible-form of nitric oxide synthase (iNOS) (11). Under hypoxic conditions, these cells generate NO (35), which may play a critical role in the pathogenesis of the above diseases. Changes in the mitochondrial membrane potential (3) and the inhibition of general respiratory status of mitochondria (Fig. 3) by cocaine could cause a situation similar to hypoxia, in terms of the inability of oxygen to be reduced to water in astroglial cells. Thus, there is a possibility that cocaine-treated astroglial cells could play a key role in the regulation of NO production. In the present report, we investigated if cocaine alters iNOS activity for NO production. Contrary to earlier reports (7), we did not observe either stimulation or inhibition of NO in these cells (Fig. 2A). This observation implies that in astroglial cells cocaine toxicity was not exerted through the NO pathway. In separate studies that did not involve astroglial cells, we evaluated if the presence of cocaine could inhibit NO production from sodium nitroprus-side, a compound that generates nitric oxide spontaneously at physiological pH. We found that cocaine did not inhibit NO production at any concentration (data not shown). From these studies, it was obvious that cocaine did not have any direct role on NO production in astroglial cells.

Respiratory measurements by a Clark oxygen electrode have several disadvantages (36–39). We, therefore, preferred to evaluate the mitochondrial respiration by the MTS assay (4,14). The dose-dependent decrease in respiratory status (Fig. 3) of astroglial cells clearly indicates that cocaine interacts with the mitochondria and impairs the energy metabolism. The higher ED_50_ value (6.153 mM) of mitochondrial respiration, compared to the LC_50_ (4.717 mM) of cell cytotoxicity, suggests that cell death was not solely due to mitochondrial damage; because in such event, cells could still survive anaerobically for some time in cultures (4).

Cocaine is known to inhibit the synthesis of several macromolecules in the cells (40,41). In agreement with these reports, we observed that cocaine decreased the total cellular protein levels in astroglial cells (Fig. 4). This inhibition was shown earlier as the direct consequence of cocaine’s interaction with ribosomes on the rough endoplasmic reticulum (42). The depletion of the protein amount in our study is correlated with the decreased cell viability (Fig. 1).

Finally, we tested cocaine’s influence to small, brine shrimp larvae. We performed this bioassay to assess whether cocaine toxicity is specific or general. So far, no pharmacological study of cocaine on shrimp larvae has been reported. We observed that acute (1–2 h) cocaine treatment did not impair the free-swimming ability of shrimp, while chronic exposure (24 h) weakened those surviving. Comparison of sublethal doses of the shrimp assay (ED_50_, 2.41 mM) (Fig. 5) and of the astroglial cells (LC_50_, 4.717 mM) (Fig. 1) indicates that live shrimp larvae are more sensitive to cocaine toxicity. The higher sensitivity of shrimp larvae could be due to their primitive nervous system. From the response of shrimp larvae and astroglial cells, it appears that cocaine toxicity is non-specific.

In conclusion, we demonstrated that cocaine treatment did not induce NO production in astroglial cells. We also showed that cocaine not only interfered with mitochondrial respiration but also caused depletion in the total protein levels in the cells. Our study highlights that the death of astroglial cells by cocaine treatment was not primarily due to any single factor but to the combined contribution from several factors like decrease in the general respiratory status and the total protein levels, showing cocaine’s generalized toxicity to the cells. Cocaine is a lipophilic compound, and readily crosses the blood brain barrier *in vivo*. Based on the high abundance of astroglial cells in the central nervous system, it appears that these cells could be the earliest targets of cocaine. This premise, however, needs further investigation *in vivo*. Currently, studies are under way in animal models.

## Abbreviations:

BCA: bicinchoninic acid;
CNS: central nervous system;
EDTA: ethylenediaminetetraacetic acid;
ED_50_: effective dose;
FBS: fetal bovine serum;
LC_50_: lethal concentration;
iNOS: inducible-form of nitric oxide synthase;
MTS: 3-(4,5-dimethylthiazol-2-yl)-5(3-carboxymethonyphenol)-2-(4-sulfophenyl)-2H-tetrazolium;
NED: N-(1-naphthyl)-ethylenediamine;
NO: nitric oxide;
PBS: pho- sphate-buffered saline
